# Asymmetric Switch Costs in Numeral Naming and Number Word Reading: Implications for Models of Bilingual Language Production

**DOI:** 10.3389/fpsyg.2015.02011

**Published:** 2016-01-25

**Authors:** Michael G. Reynolds, Sophie Schlöffel, Francesca Peressotti

**Affiliations:** ^1^Visual Cognition Lab, Department of Psychology, Trent UniversityPeterborough, ON, Canada; ^2^Basque Center on Cognition, Brain and LanguageSan Sebastian, Spain; ^3^Dipartimento di Psicologia Dello Sviluppo e Della Socializzazione, Universita degli Studi di PadovaPadova, Italy

**Keywords:** bilingualism, speech production, lexical decision, language switching, language comprehension, controlled processing

## Abstract

One approach used to gain insight into the processes underlying bilingual language comprehension and production examines the costs that arise from switching languages. For unbalanced bilinguals, asymmetric switch costs are reported in speech production, where the switch cost for L1 is larger than the switch cost for L2, whereas, symmetric switch costs are reported in language comprehension tasks, where the cost of switching is the same for L1 and L2. Presently, it is unclear why asymmetric switch costs are observed in speech production, but not in language comprehension. Three experiments are reported that simultaneously examine methodological explanations of task related differences in the switch cost asymmetry and the predictions of three accounts of the switch cost asymmetry in speech production. The results of these experiments suggest that (1) the type of language task (comprehension vs. production) determines whether an asymmetric switch cost is observed and (2) at least some of the switch cost asymmetry arises within the language system.

## Introduction

How do individuals who speak more than one language (hereafter referred to as bilinguals) coordinate their language systems so as to produce continuous speech in a single language and yet switch to an appropriate language as required? One approach used to investigate this issue examines the costs that arise when bilinguals switch between languages (e.g., Von Studnitz and Green, 1997; Meuter and Allport, 1999; Thomas and Allport, 2000; Costa and Santesteban, 2004; Orfanidou and Sumner, 2005; Peeters et al., 2014). This approach utilizes the methods and reasoning developed in the task switching literature in order to gain insight into the processes underlying control over the bilingual language system (e.g., Allport et al., 1994; Rogers and Monsell, 1995; see Kiesel et al., 2010; Vandierendonck et al., 2010, for recent reviews). These methods examine whether there is a cost to switching languages on a trial-by-trial basis in response to discrete stimuli by comparing performance on trials where a language repeats (non-switch trials) to performance on trials where the language changes (switch trials). Evidence for control comes from worse performance on switch trials (longer response times and/or increased error rates) compared to non-switch trials.

One often reported finding in the context of language switching is that for unbalanced bilinguals the cost of switching to a stronger language (L1) is larger than the cost of switching to a weaker language (L2). This switch cost asymmetry was initially reported by Meuter and Allport (1999) and has since been replicated in a number of studies (e.g., Costa and Santesteban, 2004; Costa et al., 2006; Finkbeiner et al., 2006; Philipp et al., 2007; Verhoef et al., 2009; Macizo et al., 2012; Peeters et al., 2014). Presently, the switch cost asymmetry in unbalanced bilinguals is attributed to differences in relative language strength (Grainger and Beauvillain, 1987; Dijkstra and Van Heuven, 1998; Green, 1998; Van Heuven et al., 1998; Meuter and Allport, 1999; Costa and Santesteban, 2004; Finkbeiner et al., 2006; Peeters et al., 2014). Consistent with these accounts, Costa and colleagues (Costa and Santesteban, 2004; Costa et al., 2006) rule out absolute language strength, age of L2 acquisition, and language similarity as factors that give rise to the switch cost asymmetry. Furthermore, the asymmetry is reduced after extended practice (Meuter and Allport, 1999) and when languages are balanced in strength such that larger differences in relative proficiency are required in order to observe asymmetric switch costs when individuals are exceptionally proficient in two languages, to the point where highly proficient bilinguals will not show a switch cost asymmetry for unbalanced languages (Costa and Santesteban, 2004; Costa et al., 2006; Martin et al., 2013).

If the switch cost asymmetry in unbalanced bilinguals arises from differences in the relative strength of the two languages, then it seems to follow that asymmetric switch costs will be observed in any task where the two languages are unbalanced in strength. However, as can be seen in Table 1, there are many studies with unbalanced bilinguals that report symmetric switch costs, inconsistent with relative language strength being the sole determining factor for a switch cost asymmetry. A quick examination of Table 1 reveals one possible source of such inconsistencies, namely the type of task. In general, asymmetric switch costs tend to be observed when the task is speech production and symmetric switch costs tend to be observed when language comprehension tasks are used (e.g., lexical decision or semantic categorization). Consistent with this possibility speech production and visual language comprehension differ in at least three important ways that could explain the different patterns of switch costs. First, different types of stimuli are used. Typically, speech production studies involve object naming whereas language comprehension studies use written words. This distinction is critical because objects (and numerals) are encoded differently than words (e.g., Humphreys et al., 1999; Damian, 2004). For instance, the visual representations of objects and words are thought to be stored in two separate lexicons: a pictogen system for objects (e.g., Humphreys et al., 1999; Coltheart, 2004) and an orthographic lexicon for words (e.g., Coltheart et al., 2001; Perry et al., 2007). Second, speech production relies on the retrieval of a phonological-lexical representation for production, whereas comprehension tasks such as lexical decision and semantic categorization rely on a search of orthographic-lexical and semantic information for a binary (yes/no) response. Finally, evidence suggests that language comprehension tasks such as lexical decision are more susceptible to decision processes than naming tasks (Chumbley and Balota, 1984).

**Table 1:** **A summary of experiments examining switch costs as a function of task, predictability of switches, stimulus type, bilingual proficiency, and language strength**.

**Paper**	**Exp**.	**Switch costs**	**Task**	**Predictability**	**Stimuli**	**Proficiency**	**Languages**
Christoffels et al., 2007	1	Symmetric	Naming	Random	Pictures	Unbalanced	Unbalanced L1 vs. L2
Costa and Santesteban, 2004	1	Asymmetric	Naming	Random	Pictures	Unbalanced	Unbalanced L1 vs. L2
Costa and Santesteban, 2004	2	Symmetric	Naming	Random	Pictures	Highly proficient	Balanced L1 vs. L2
Costa and Santesteban, 2004	3	Symmetric	Naming	Random	Pictures	Highly proficient	Balanced L1 vs. L2
Costa and Santesteban, 2004	4	Symmetric	Naming	Random	Pictures	Highly proficient	Unbalanced L1 vs. L3
Costa and Santesteban, 2004	5	Symmetric	Naming	Random	Pictures	Highly proficient	Balanced L1 vs. L2
Costa and Santesteban, 2004	5	Symmetric	Naming	Random	Pictures	Highly proficient	Balanced L1 vs. L2
Costa et al., 2006	1	Symmetric	Naming	Random	Pictures	Highly proficient	Balanced L1 vs. L2
Costa et al., 2006	2	Symmetric	Naming	Random	Pictures	Highly proficient	Unbalanced L2 vs. L3
Costa et al., 2006	3	Asymmetric	Naming	Random	Pictures	Highly proficient	Unbalanced L3 vs. L4
Costa et al., 2006	4	Asymmetric	Naming	Random	Pictures	Highly proficient	Unbalanced L1 vs. New
Declerck et al., 2012	1	Symmetric	Naming	Random	Numerals	Unbalanced	Unbalanced L1 vs. L2
Declerck et al., 2012	1	Symmetric	Naming	Random	Pictures	Unbalanced	Unbalanced L1 vs. L2
Fink and Goldrick, 2015	1	Symmetric	Naming	Random	Digits	Highly proficient	Balanced L1 vs. L2
Fink and Goldrick, 2015	1	Asymmetric	Naming	Random	Digits	Unbalanced	Unbalanced L1 vs. L2
Jackson et al., 2001	1	Asymmetric	Naming	Predictable	Numerals	Unbalanced	Unbalanced L1 vs. L2
Jackson et al., 2004	1	Asymmetric	Parity	Predictable	Number words	Unbalanced	Unbalanced L1 vs. L2
Macizo et al., 2012	1	Asymmetric	Naming	Random	Words	Unbalanced	Unbalanced L1 vs. L2
Macizo et al., 2012	2	Symmetric	Categorization	Random	Words	Unbalanced	Unbalanced L1 vs. L2
Macizo et al., 2012	3	Symmetric	Categorization	Random	Words	Unbalanced	Unbalanced L1 vs. L2
Martin et al., 2013	1	Asymmetric	Naming	Random	Pictures	Highly proficient	Balanced L1 vs. L2
Martin et al., 2013	1	Symmetric	Naming	Random	Pictures	Highly proficient	Unbalanced L1 vs. L3
Martin et al., 2013	1	Asymmetric	Naming	Random	Pictures	Highly proficient	Unbalanced L1 vs. L3
Meuter and Allport, 1999	1	Asymmetric	Naming	Random	Numerals	Unbalanced	Unbalanced L1 vs. L2
Orfanidou and Sumner, 2005	1	Symmetric	LD ⋆	Predictable	Words	Unbalanced	Unbalanced L1 vs. L2
Orfanidou and Sumner, 2005	2	Symmetric	LD ⋆	Predictable	Words	Unbalanced	Unbalanced L1 vs. L2
Philipp et al., 2007	1	Asymmetric	Naming	Random	Numerals	Unbalanced	Unbalanced L1 vs. L2
Philipp et al., 2007	1	Asymmetric	Naming	Random	Numerals	Unbalanced	Unbalanced L1 vs. L2
Thomas and Allport, 2000	1	Symmetric	LD 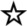	Random	Words	Unbalanced	Unbalanced L1 vs. L2
Thomas and Allport, 2000	2	Symmetric	LD ⋆	Predictable	Words	Unbalanced	Unbalanced L1 vs. L2
Thomas and Allport, 2000	3	Symmetric	LD ⋆	Predictable	Words	Unbalanced	Unbalanced L1 vs. L2
Verhoef et al., 2009	1 ♢	Symmetric	Naming	Random	Pictures	Unbalanced	Unbalanced L1 vs. L2
Verhoef et al., 2009	1 ♦	Asymmetric	Naming	Random	Pictures	Unbalanced	Unbalanced L1 vs. L2
Von Studnitz and Green, 1997	1	Symmetric	LD ⋆	Predictable	Words	Unbalanced	Unbalanced L1 vs. L2
Von Studnitz and Green, 1997	2	Symmetric	LD 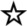	Predictable	Words	Unbalanced	Unbalanced L1 vs. L2
Von Studnitz and Green, 2002	1	Symmetric	Categorization	Predictable	Words	Unbalanced	Unbalanced L1 vs. L2

⋆, Language exclusive instructions; 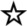, language inclusive instructions; ♦, short cue duration; ♢, long cue duration; LD, Lexical Decision.

Although it is possible that the presence and absence of a switch cost asymmetry in unbalanced bilinguals is due to the type of task, it is necessary to rule out other competing explanations. As can be seen in Table 1, two other factors that are confounded with the presence/absence of the switch cost asymmetry are (1) the type of stimuli (univalent vs. bivalent) and (2) the predictability of the language switches.

The present experiments serve two goals. The first goal is to assess whether the presence of the switch cost asymmetry for unbalanced bilinguals in speech production tasks and its absence in language comprehension tasks, such as lexical decision, are a consequence of methodological differences or differences in how languages are activated during speech production and language comprehension tasks. The second goal is to discriminate between accounts of the switch cost asymmetry. In order to accomplish these goals, Experiments 1 and 2 assess whether naming yields symmetric switch costs like lexical decision when methodological differences are eliminated. Finally, Experiment 3 assesses whether languages are activated differently in naming and lexical decision by examining whether stimulus valence influences switch costs under mixed list presentation conditions in the naming task^1^.

## Experiments 1A and 1B

One factor that may determine whether a switch cost asymmetry is observed is the type of stimuli. The stimuli used in language switching experiments differ in two important ways. The first concerns stimulus valence, which refers to the correspondence between a stimulus and a task. Bivalent stimuli are those that are used to respond in both tasks, whereas univalent stimuli are those that correspond to a single task. The second concerns how likely it is that a stimulus belongs to a given language. In speech production, the stimuli tend to be numerals or pictures, which are named in both languages during an experiment (bivalent) and do not contain language specific information. In contrast, in lexical decision or semantic categorization studies, the stimuli tend to be different sets of written words for each language (univalent) and the words tend to be unique to one language (contain language specific information). It is therefore possible that univalent words containing language specific information might eliminate the switch cost asymmetry because they trigger the appropriate language more directly than pictures and numerals, and therefore do not require the same level of non-target language inhibition (Grainger et al., 2010). Indeed, Peeters et al. (2014) argued that words automatically activate the corresponding language. Consistent with this interpretation, studies that have used univalent stimuli tend to show symmetric switch costs (but see Jackson et al., 2004; Macizo et al., 2012).

Experiments 1A and 1B assess whether the switch cost asymmetry arises when bivalent stimuli (i.e., numerals) are used and is absent when univalent stimuli (i.e., written words that appear in only one language) are used. If the switch cost asymmetry were eliminated for univalent stimuli, then this would suggest that switch costs in language comprehension and language production tasks arise from the same processes.

Examining how the switch cost asymmetry is affected by stimulus valence also has implications for accounts of language switching in speech production. For instance, Finkbeiner et al.'s (2006) response selection account of the switch cost asymmetry predicts that the switch cost asymmetry should be eliminated when univalent stimuli are used. According to this account, when stimuli are bivalent (associated with both languages), both language systems generate a viable response. On switch trials, the response criteria change in order to select the response generated by the appropriate language, yielding a switch cost. Asymmetric switch costs arise because on a subset of switch trials the easier L1 response becomes available before the response selection criteria have been updated and is therefore rejected. This creates an additional cost on L1 switch trials because the response must be regenerated. Unlike bivalent stimuli, univalent items only activate a response in one language; consequently, when univalent stimuli are used a single response selection criterion can be used eliminating the need to switch response criteria. Data in support of this account are mixed. Consistent with the response selection account, Finkbeiner et al. (2006) reported that the switch cost asymmetry is eliminated when univalent stimuli are used. However, Peeters et al. (2014) reported a switch cost asymmetry for univalent items. Unfortunately, in both cases, language switching was confounded with task switching, which complicates interpretation of their data. For instance in Finkbeiner et al. (2006), numerals were named in both languages whereas pictures (Experiment 1) and dots (Experiment 2) were used as univalent stimuli (only named in one language). Critically, there was no cost to switching languages for the univalent items, consistent with the response selection account. However, naming pictures and numerals is unlikely to require the same cognitive processes (see Abutalebi and Green, 2007). Indeed, evidence suggests that language switching differs when numerals and pictures are used as stimuli (Declerck et al., 2012). It is therefore possible that language switch costs were not observed for univalent items because, irrespective of whether a language switch was taking place, switching from numerals to pictures (as in their Experiment 1) constituted a task switch. A similar situation occurred in Peeters et al. (2014), where subjects switched from making binary decisions about words (e.g., lexical decision) to naming pictures. Again, a task switch corresponded with switching to the univalent stimuli, rendering interpretation of their data difficult. One finding that seems to be inconsistent with the response selection account has been reported by Macizo et al. (2012). In this study, different sets of words (univalent stimuli) were named for each language. Inconsistent with Finkbeiner et al.'s (2006) response selection account, univalent stimuli produced an asymmetric switch cost.

In the present experiments, we explored these issues by having unbalanced bilinguals alternate between naming (bivalent) numerals in L1 and L2 in one block of trials and (univalent) number words in L1 and L2 in another block of trials. Language switches were random in order to match other studies that have observed a switch cost asymmetry in speech production (e.g., Meuter and Allport, 1999; Costa and Santesteban, 2004; Costa et al., 2006; Macizo et al., 2012). Stimulus valence was blocked because according to Finkbeiner et al. a univalent item should only yield a response in one language permitting a single response criterion to be used whereas under mixed list conditions different response criteria may be used for univalent and bivalent stimuli. Therefore, Finkbeiner et al.'s (2006) response selection account predicts that neither switch costs nor a switch cost asymmetry should be observed for univalent stimuli under blocked list conditions.

### Method

#### Participants

Forty undergraduate students (32 female, 8 male) participated in the experiment^2^. The students received course credit in an eligible psychology course as compensation. All participants had normal or corrected to normal vision. This study was carried out in accordance with the recommendations of Tri-Council Policy Statement: Ethical Conduct for Research Involving Humans, Natural Sciences and Engineering Council of Canada, with written informed consent from all subjects in accordance with the Declaration of Helsinki.

Twenty participants who were students at the University of Padova, Italy, participated in Experiment 1A. They reported Italian as their stronger first language (L1) and English as their weaker second language (L2). They began studying English at a variable age (between 5 and 13 years old; mean = 6.2). All 20 participants studied English as a second language during the 5 years of the High School. Twelve of the participants had traveled to an English speaking country in the last 5 years. They self-evaluated their proficiency in English as 3.51 on a Likert 7-point scale (1 very low—7 excellent).

Twenty participants who were students at Trent University, Canada, participated in Experiment 1B. They reported English as their stronger first language (L1) and French as their weaker second language (L2). All participants had studied French in school for a minimum of 6 years (mean = 8.45) as per Ontario education curriculum (Ministry of Education and Training, 1998, 1999). Seventy percent of the participants reported being able to produce and comprehend simple instructions and written material in L2 (Treasury Board of Canada Secretariat, 2012). None described themselves as perfectly matched in English and French.

#### Stimuli

Stimuli were either numerals (bivalent) or number words (univalent) ranging from 1 to 9. In Experiment 1B, the cognate *six*, which is the same word in both French and English, was replaced with 10 (see also Jackson et al., 2004). All stimuli were presented in a white 16-pt. Times New Roman font against a black background. The words were presented in lower case letters. Numerals subtended 0.6° by 0.6° visual angle. Number words subtended 0.6° visual angle vertically and from 1.7° to 3.4° horizontally.

The language cue consisted of a box subtending 6.9° × 6.9° degrees visual angle that surrounded the location of the target. The interior of the box matched the background color (black). The border of the box was 3 pixels thick (approximately 0.1° visual angle) and was always visible. The color of the box was used to indicate the appropriate response language on a given trial. Standard EPrime colors were used (Experiment 1A: green for Italian, blue for English; Experiment 1B: red for English, blue for French).

#### Apparatus

The experiment was conducted using a computer running MicroSoft Windows XP operating system. Stimulus presentation and data collection were controlled using EPrime 2.0 software. Vocal responses were collected using a PST Response Box with a voice-key assembly.

#### Procedure

Participants were tested individually in a sound attenuated, dimly lit room. They were seated approximately 50 cm from the computer monitor with the microphone placed directly in front of them. Written instructions were presented on the computer screen in the participants' native language (L1). Participants were required to name each stimulus as quickly and accurately as possible in the appropriate language. Depending on the block, participants were informed that numerals or number words would appear on the computer screen. Order of block presentation (numerals vs. words) was counterbalanced across subjects where the assignment of subject to counterbalance was determined pseudorandomly based on the order in which they were tested.

Within each block, subjects were presented with 9 set lists of pseudorandomized trials. Each list consisted of 46 trials with predetermined slots for switch and non-switch trials. *Switch* trials were preceded by a stimulus to be named in the other language and *non-switch* trials were preceded by a same-language stimulus. In order to match the conditions under which the switch cost asymmetry was initially observed (Meuter and Allport, 1999; Costa and Santesteban, 2004; Costa et al., 2006), the lists were constructed so that the probability of a switch was 0.3 and a non-switch was 0.7. The run length ranged from 1 to 7. The assignment of language to the first trial of a list was counterbalanced across subjects. The first trial in each list was then coded as a null switch trial and excluded from subsequent analyses. The assignment of a stimulus on a given trial was determined randomly without replacement. The same stimulus was permitted to occur on consecutive trials. The number of switches per list ranged from 11 to 20. The order of lists was randomized for each subject, with the first list treated as practice trials. Overall, there were 256 switch and 464 non-switch experimental trials per subject.

Each trial began with the presentation of the box cue in a neutral silver color. After 250 ms, the target stimulus was presented inside the box cue simultaneously with the color of the box cue changing to indicate the response language. The target stimulus and box cue remained visible until a vocal response was made. As soon as a vocal response was initiated, the target stimulus disappeared and the box color changed to white. This screen remained while the experimenter coded the vocal response via button press as correct, incorrect (incorrect language vs. within-language error), or a voice-key failure. Once the response was coded, a new stimulus appeared. Participants were given the opportunity to take a break every 46 trials. The experiment took approximately 40 min to complete.

### Results

The mean correct response time (RT) and percentage error data were analyzed separately using analysis of variance (ANOVA) with Language (L1 vs. L2), Stimulus Type (Numerals vs. Words), and Trial Type (Switch vs. Non-switch) as within subjects factors and Experiment (Italian-English: 1A vs. English-French: 1B) as a between subjects factor. Mean response times and percentage error are displayed in Table 2.

**Table 2:** **Mean RT (ms) and percentage errors from Experiments 1A and 1B (random switches) as a function of stimulus type (numerals vs. words), trial type (switch vs. non-switch) and language (L1 vs. L2)**.

	**Response time**				**Percentage error**			
	**Numerals**		**Words**		**Numerals**		**Words**	
	**L1**	**L2**	**L1**	**L2**	**L1**	**L2**	**L1**	**L2**
**ITALIAN/ENGLISH BILINGUALS**								
Switch	674	646	500	499	5.3	5.0	2.1	2.3
Non-switch	592	608	468	488	3.6	4.3	1.0	1.6
Cost	82	38	32	11	1.7	0.6	1.1	0.7
**ENGLISH/FRENCH BILINGUALS**								
Switch	731	753	530	532	7.8	7.1	1.3	1.3
Non-switch	659	710	516	526	3.7	4.3	1.0	1.6
Cost	72	43	14	6	4.2	2.8	0.2	−0.4

#### RT

Prior to analyzing the RT data, trials with incorrect responses (3.05%) or voice-key errors (0.59%) were first removed. RTs to correct responses were subjected to a recursive trimming procedure in which the criterion cutoff for outlier removal was established independently for each condition for each subject by reference to the sample size in that cell (Van Selst and Jolicoeur, 1994). This resulted in the removal of an additional 1.57% of the data.

As can be seen in Table 2, there was a main effect of stimulus type where participants took 165 ms longer to respond to numerals compared to number words, *F*_(1, 38)_ = 420.1, *p* < 0.001, *MSE* = 5127, η_p_^2^ = 0.92. Overall, the effect of stimulus type is comparable with other studies that have manipulated stimulus valence by changing the orthographic characteristics of words in lexical decision (e.g., 160 ms in Thomas and Allport, 2000 and 180 ms in Orfanidou and Sumner, 2005). Consistent with our participants being unbalanced bilinguals, there was a main effect of language where responses in L1 were faster than responses in L2 [*F*_(1, 38)_ = 6.58, *p* < 0.05, *MSE* = 1759, η_p_^2^ = 0.15]. Consistent with participants adjusting processing in response to changes in language, there was a main effect of trial type where switch trials were 37 ms slower than non-switch trials, *F*_(1, 38)_ = 126.2, *p* < 0.001, *MSE* = 885, η_p_^2^ = 0.77. There was an interaction between trial type and stimulus type where the size of the switch cost was larger for numerals (77 ms) than for number words (16 ms), *F*_(1, 38)_ = 82.0, *p* < 0.001, *MSE* = 449, η_p_^2^ = 0.68. This replicates Orfanidou and Sumner (2005) finding in lexical decision and extends it to speech production.

As expected, there was an interaction between trial type and language where the cost of switching languages was larger for L1 (50 ms) than for L2 (24 ms), replicating the switch cost asymmetry obtained by Meuter and Allport (1999; see also Costa and Santesteban, 2004; Costa et al., 2006; Verhoef et al., 2009; Macizo et al., 2012), *F*_(1, 38)_ = 33.0, *p* < 0.001, *MSE* = 410, η_p_^2^ = 0.46. Also, the three way interaction between stimulus type, trial type and language was significant, *F*_(1, 38)_ = 8.13, *p* < 0.01, *MSE* = 298, $\eta_{\text{p}}^{2}=0.18$. This suggests that the switch cost asymmetry is affected by stimulus valence, since the asymmetry was smaller for the number words (15 ms) compared to the numerals (37 ms).

Since the main goal of the present experiment was to assess whether the switch cost asymmetry would be eliminated by the use of univalent stimuli, separate ANOVAs were performed for numerals and number words with Language (L1 vs. L2) and Trial Type (switch vs. non-switch) as factors. Both analyses showed significant interactions between the two factors [Numerals: *F*_(1, 38)_ = 24.4, *p* < 0.001, *MSE* = 560.67, η_p_^2^ = 0.39; Words: *F*_(1, 38)_ = 15.23, *p* < 0.001, *MSE* = 147.28, η_p_^2^ = 0.29], suggesting that for both numerals and number words, the switch cost was larger in L1 than in L2. The small switch cost observed for the univalent stimuli in L2 was also reliable [*F*_(1, 38)_ = 9.54, *p* < 0.01, *MSE* = 151, η_p_^2^ = 0.20] and was unaffected by Experiment (*F* < 1).

There was a main effect of Experiment, where responses in Experiment 1A were 60 ms faster than the responses in Experiment 1B, *F*_(1, 38)_ = 6.56, *p* < 0.05, *MSE* = 44271, η_p_^2^ = 0.18. Experiment interacted with stimulus type, where the effect of stimulus type was smaller for the Italian/English bilinguals in Experiment 1A (141 ms) compared to the English/French bilinguals in Experiment 1B (187 ms), *F*_(1, 38)_ = 8.25, *p* < 0.01, *MSE* = 5127, η_p_^2^ = 0.18 Experiment also interacted with language where the effect of language was primarily due to Experiment 1B [Italian-English: 558 vs. 560 ms; English-French: 609 vs. 630 ms; *F*_(1, 38)_ = 4.12, *p* < 0.05, *MSE* = 1759, η_p_^2^ = 0.14]. Finally, there was a three way interaction between experiment, language and stimulus type, *F*_(1, 38)_ = 12.8, *p* < 0.05, *MSE* = 824, η_p_^2^ = 0.25. The L1 advantage for English-French bilinguals was larger for numerals (37 ms) than for words (6 ms), *F*_(1, 19)_ = 7.142, *p* < 0.05, *MSE* = 1283, η_p_^2^ = 0.273. The advantage for words was not reliable (*F* < 1). In contrast, the L1 advantage for Italian-English bilinguals was smaller for numerals (–2 ms) than for words (10 ms), *F*_(1, 19)_ = 6.727, *p* < 0.05, *MSE* = 365, η_p_^2^ = 0.261. The advantage for numerals was not reliable (*F* < 1).

#### Percent error

There was nothing in the error data that compromised the interpretation of the RT data. There was a main effect of Trial Type [*F*_(1, 38)_ = 34, *p* < 0.001, *MSE* = 0.001; η_p_^2^ = 0.47], reflecting a higher error rate for switch (4%) than for non-switch (2.4%) trials and a main effect of Stimulus Type [*F*_(1, 38)_ = 73.85, *p* < 0.001, *MSE* = 0.001, η_p_^2^ = 0.66], reflecting more errors for numerals than for words (4.9 vs. 1.5%). There was an interaction between Stimulus Type and Experiment, where the difference between numerals and words was larger in Experiment 1A (5.7 vs. 1.3%) than in Experiment 1B [4.2 vs. 1.7%, *F*_(1, 38)_ = 5.47, *p* = 0.025, *MSE* = 0.001, η_p_^2^ = 0.13]. There was an interaction between Stimulus Type and Trial Type where the switch cost was larger for numerals (switch: 6.3%, non-switch: 3.6%) than for words [switch: 1.7%, non-switch: 1.3%; *F*_(1, 38)_ = 11.2, *p* = 0.002, *MSE* = 0.001, η_p_^2^ = 0.23]. There was also an interaction between Trial Type and Experiment where the switch cost was larger in Experiment 1A than in Experiment 1B [*F*_(1, 38)_ = 4.8, *p* = 0.034, *MSE* = 0.001, η_p_^2^ = 0.11].

### Discussion

Given that the presence/absence of the switch cost asymmetry reported in previous studies largely co-varied with stimulus valence, Experiments 1A and 1B assessed whether the switch cost asymmetry in speech production is present for (bivalent) numerals but absent for (univalent) number words. The observation of a switch cost asymmetry in speech production, despite the use of univalent stimuli (i.e., number words) is inconsistent with bivalent stimuli (e.g., numerals and pictures) being required to observe the switch cost asymmetry. The absence of a switch cost asymmetry in lexical decision studies therefore cannot be due to the use of written words.

The observation that the switch cost asymmetry was not eliminated for univalent stimuli is also inconsistent with Finkbeiner et al.'s (2006) response selection account of the switch cost asymmetry. According to this account, univalent stimuli should not yield a switch cost when stimulus valence is blocked because a single response selection criterion can be used for all of the univalent stimuli. Given that each univalent word was tied to a response in only one language, there was no need to change the response criteria across language switch trials. The response selection account of switch costs therefore not only predicts the absence of a switch cost asymmetry, but also the absence of a switch cost. Neither of these predicted outcomes were observed.

## Experiments 2A and 2B

As can be seen in Table 1, a second factor that co-varies with the presence/absence of the switch cost asymmetry is switch predictability. Studies that report a switch cost asymmetry have typically used random switches between categories (similar to Meuter and Allport, 1999). In contrast, studies that do not report a switch cost asymmetry (e.g., Von Studnitz and Green, 1997; Thomas and Allport, 2000) have tended to use predictable switches between languages, based on a variation of Rogers and Monsell's (1995) alternating runs paradigm in which switches occur in a predictable AABB pattern. It is therefore possible that random switches between languages are required (necessary, but not sufficient) in order to observe a switch cost asymmetry. For instance, the switch cost asymmetry may not be observed when switches are predictable because advanced knowledge of the language switch provides the opportunity to endogenously prepare for the language switch prior to the presentation of the stimulus^3^. Otherwise, it might be that symmetric switch costs depend on the conjunction of predictable switches and univalent stimuli. The aim of the present experiment was to test these hypotheses. Similar to Experiments 1A and 1B, participants in Experiment 2A were Italian / English bilinguals, and participants in Experiment 2B were English/French bilinguals. Again, the task was naming (bivalent) numerals and (univalent) number words, with valence assigned to different blocks of trials as in the previous experiment. Following Rogers and Monsell's (1995) alternating runs paradigm, the assignment of language for responding followed an AA/BB pattern (a run length of 2).

### Method

#### Participants

Thirty-six undergraduate students (32 female, 4 male) participated in the experiment. They all received credit in an eligible psychology course as compensation. All participants had normal or corrected to normal vision.

Sixteen participants were students from the University of Padova and participated in Experiment 2A. They were unbalanced Italian-English bilinguals with Italian as stronger first language (L1) and English as their weaker second language (L2). They met the same criteria as subjects in Experiment 1A.

Twenty participants were undergraduate students at Trent University and participated in Experiment 2B. They were unbalanced English-French bilinguals with English as their stronger first language (L1) and French as their weaker second language (L2). They met the same criteria as participants Experiment 1B.

#### Stimuli

Target stimuli were identical to Experiments 1A and 1B. Following Rogers and Monsell (1995), a 2 × 2 grid was used to help subjects keep track of the predictable AABB pattern^4^. Each square in the grid subtended 6.9° by 6.9° visual angle as in Experiments 1A and 1B.

#### Apparatus

The apparatus was identical to Experiments 1A and 1B.

#### Procedure

The procedure was similar to Experiments 1A and 1B. Once again, the experiment consisted of two blocks, with numerals being presented in one block and number words presented in the other. The assignment of Stimulus Type to block was counterbalanced pseudorandomly across subjects based on the order in which they were tested. Within each block, subjects were presented with 9 lists of 44 trials with a predictable switch after two consecutive trials in the same language (i.e., a run length of 2). As in Experiments 1A and 1B, the assignment of stimulus to trial was determined randomly without replacement. The first list in each block was treated as practice.

The 2 × 2 display grid was visible throughout the presentation of a list of trials. A trial started with an empty display grid. After 250 ms, the target stimulus was presented in the center of one of the four boxes. The stimulus remained visible until a vocal response was made. The accuracy of the vocal response was then coded via button press by the researcher before the beginning of the next trial. The location of a target on successive trials moved to the adjacent clockwise location in the grid. Adjacent horizontal locations always corresponded to a single language and the assignment of language to position (top vs. bottom) was determined randomly for each subject. Participants were given the opportunity to take a break every 44 trials. The experiment took approximately 40 min to complete.

### Results

The data were analyzed in the same way as Experiment 1. Mean response latencies and accuracies are displayed in Table 3.

**Table 3:** **Mean RT (ms) and percentage errors from Experiments 2A and 2B (predictable switches) as a function of stimulus type (numerals vs. words), trial type (switch vs. non-switch) and language (L1 vs. L2)**.

	**Response time**				**Percentage error**			
	**Numerals**		**Words**		**Numerals**		**Words**	
	**L1**	**L2**	**L1**	**L2**	**L1**	**L2**	**L1**	**L2**
**ITALIAN/ENGLISH BILINGUALS**								
Switch	660	661	512	525	6.9	7.1	2.1	2.0
Non-switch	549	600	472	499	4.5	5.0	1.2	0.7
Cost	111	61	40	26	2.4	2.1	0.9	1.3
**ENGLISH/FRENCH BILINGUALS**								
Switch	693	727	507	518	7.2	7.8	2.0	0.9
Non-switch	594	666	481	500	3.1	4.1	0.5	0.5
Cost	99	61	26	18	4.1	3.7	1.5	0.4

#### RT

Prior to analyzing the RT data, trials with incorrect responses (2.9%) or voice-key errors (0.75%) were removed. RTs to correct responses were subjected to the same recursive trimming procedure used in Experiment 1 (Van Selst and Jolicoeur, 1994). This resulted in the removal of an additional 2.03% of the data.

As can be seen in Table 3, there was a main effect of stimulus type where participants took 142 ms longer to name numerals than number words, *F*_(1, 34)_ = 128.1, *p* < 0.001, *MSE* = 11,149, $\eta_{\text{p}}^{2}=0$.79. The effect of stimulus valence is comparable in magnitude to Experiment 1 [*F*_(1, 74)_ = 1.61, *p* = 0.209, *MSE* = 8994, $\eta_{\text{p}}^{2}=0$.02]^5^ and with previous studies that have used the lexical decision task (Von Studnitz and Green, 1997; Thomas and Allport, 2000; Orfanidou and Sumner, 2005). Consistent with our participants being unbalanced bilinguals, there was a main effect of language where L1 responses were 28 ms faster than L2 responses, *F*_(1, 34)_ = 8.02, *p* < 0.05, *MSE* = 2684, $\eta_{\text{p}}^{2}=0$.32.

Once again, there was a main effect of trial type where participants took longer to respond on switch trials compared to non-switch trials, yielding a 55 ms switch cost, *F*_(1, 34)_ = 212.2, *p* < 0.001, *MSE* = 1023, η_p_^2^ = 0.86. Consistent with stimulus valence influencing language selection, there was an interaction between stimulus type and trial type where the switch cost was 55 ms larger for numerals (83 ms) than for the number words (28 ms), *F*_(1, 34)_ = 103.5, *p* < 0.001, *MSE* = 525, η_p_^2^ = 0.75. This replicates the pattern observed in Experiment 1 and previously reported by Orfanidou and Sumner (2005) in lexical decision. Also consistent with stimulus valence influencing language selection, there was an interaction between language and stimulus type where L1 responses were 40 ms faster than L2 responses for numerals, but only 17 ms faster than L2 responses for number words, *F*_(1, 34)_ = 8.96, *p* < 0.05, *MSE* = 1003, η_p_^2^ = 0.21. The interaction between trial type and language was significant, where the switch costs were asymmetric, with a 28 ms larger switch cost in L1 (69 ms) than in L2 (41 ms), *F*_(1, 34)_ = 26.0, *p* < 0.001, *MSE* = 536, $\eta_{\text{p}}^{2}=0$.43. Critically, there was an interaction between trial type, language and stimulus type where the switch cost asymmetry was larger for numerals than for number words, *F*_(1, 34)_ = 12.9, *p* < 0.001, *MSE* = 389, $\eta_{\text{p}}^{2}=0$.28. This replicates the pattern observed in Experiments 1A and 1B.

Since the main goal of the Experiment 2 was to assess whether the switch cost asymmetry would be eliminated by the use of predictable switches, additional repeated-measure ANOVAs were performed separately for the numeral and number word conditions with Language (L1 vs. L2) and Trial Type (switch vs. non-switch) as factors. Inconsistent with predictable switches between languages eliminating the switch cost asymmetry, a switch cost asymmetry was observed for numerals, *F*_(1, 34)_ = 26.6, *p* < 0.001, *MSE =* 670, $\eta_{\text{p}}^{2}=0$.44, where the switch cost was 44 ms larger in L1 (105 ms) compared to L2 (61 ms), and this effect was not qualified by experiment (*F* < 1).

Inconsistent with the conjunction of predictable switches and the univalent stimuli being necessary to eliminate the switch cost asymmetry, there was a reliable switch cost asymmetry for the (univalent) number words, *F*_(1, 34)_ = 4.4, *p* < 0.05, *MSE* = 254, $\eta_{\text{p}}^{2}=0$.11. The switch cost was 11 ms larger in L1 (33 ms) compared to L2 (22 ms), and it was not qualified by experiment (*F* < 1).

No main effect of Experiment was obtained, *F*_(1, 34)_ = 1.21, *p* = 0.279, *MSE* = 40,069, $\eta_{\text{p}}^{2}=0$.03. However, there was an interaction between experiment and stimulus type where the effect of stimulus type was smaller for the Italian/English bilinguals (115 ms) compared to the English/French bilinguals (168 ms), *F*_(1, 34)_ = 4.43, *p* < 0.05, *MSE* = 11,149, $\eta_{\text{p}}^{2}=0$.11. Also, similar to Experiments 1A and 1B, there was an interaction between language, stimulus type and experiment where the language by stimulus type interaction was more pronounced in the English/French bilinguals than in the Italian/English bilinguals, *F*_(1, 34)_ = 4.47, *p* < 0.05, *MSE* = 1003, $\eta_{\text{p}}^{2}=0$.12.

#### Percent error

There were no effects in the error data that compromised the interpretation of the RT data. There was a main effect of Stimulus Type where more errors were made to numerals than to number words, *F*_(1, 34)_ = 88.4, *p* < 0.001, *MSE* = 0.001, $\eta_{\text{p}}^{2}=0$.72. There was a main effect of Trial Type where more errors were made on switch compared to non-switch trials, *F*_(1, 34)_ = 51.4, *p* = 0.001, *MSE* < 0.001, $\eta_{\text{p}}^{2}=0$.60. As in Experiment 1, there was an interaction between trial type and Experiment, *F*_(1, 34)_ = 8.12, *p* = 0.007, *MSE* < 0.001, $\eta_{\text{p}}^{2}=0$.19. However, here the switch cost was larger in Experiment 2B (English-French: 2.5%) than in Experiment 2A (Italian-English: 1%). There was also an interaction between Stimulus Type and Trial Type where the switch cost was larger for numerals (switch: 6.2%; non-switch: 3.4%) than it was for number words (switch: 1.3%; non-switch: 0.6%), *F*_(1, 34)_ = 14.2, *p* = 0.001, *MSE* = 0.001, $\eta_{\text{p}}^{2}=0$.30. No other effects were significant (*Fs* < 1.4).

### Discussion

The goal of Experiments 2A and 2B was to assess whether the absence of a switch cost asymmetry reported in previous work was due to the use of predictable switches between languages. Inconsistent with unpredictable switches being required for asymmetric switch costs, asymmetric switch costs were observed for numerals despite predictable switches between languages. Furthermore, the switch cost asymmetry was once again observed for number words, which uniquely indicated the language to be used for the response. This suggests that the absence of the switch cost asymmetry in lexical decision studies was not due to the conjunction of predictable switches and the use of univalent stimuli. Further, this second demonstration that the switch cost asymmetry is not eliminated for univalent stimuli provides additional evidence inconsistent with Finkbeiner et al.'s (2006) response selection account of language switching, which predicts that switch costs should not be observed when the response selection criteria do not need to change.

## Experiment 3

The outcomes of Experiments 1 and 2 indicate that the switch cost asymmetry is not due to methodological differences such as the type of stimuli or the predictability of switches. The data also indicate that the switch cost asymmetry in speech production is not a consequence of changing response criteria for bivalent stimuli as hypothesized by Finkbeiner et al. (2006). Instead the outcomes of Experiments 1 and 2 suggest that the switch cost asymmetry is observed in speech production because of how the languages are activated/inhibited. Converging evidence for this claim comes from two additional sources. First, stimulus valence interacted with language, which suggests that the two factors affect a common process, most likely one involved in language selection. Second, stimulus valence affected the magnitude of the switch cost asymmetry, whereby the switch cost asymmetry was smaller for the univalent stimuli than for the bivalent stimuli. This is consistent with language specific information contained in the stimulus reducing the impact of language strength on the selection process.

If the switch cost asymmetry arises from how languages are activated/inhibited then this suggests that languages are activated differently during comprehension and speech production tasks. In order to test this possibility, Experiment 3 examines how switch costs are affected by stimulus valence when the univalent and bivalent stimuli are randomly intermixed in the naming task. Previous research suggests that in the lexical decision task, switch costs are only reduced for univalent items when univalent and bivalent stimuli are presented in separate blocks, as in Experiments 1 and 2 (Orfanidou and Sumner, 2005). When univalent and bivalent items are randomly intermixed in a single block of trials, switch costs are unaffected by stimulus valence in lexical decision (Von Studnitz and Green, 1997; Thomas and Allport, 2000; Orfanidou and Sumner, 2005). If the switch cost asymmetry arises in speech production but not in language comprehension because there are fundamental differences in how languages are activated in language comprehension and speech production tasks, then this raises the possibility that stimulus valence will continue to affect switch costs in speech production when univalent and bivalent stimuli are randomly intermixed. In contrast, if stimulus valence does not affect the magnitude of the switch costs in mixed lists during speech production, then this will mirror the pattern previously obtained in lexical decision (Von Studnitz and Green, 1997; Thomas and Allport, 2000; Orfanidou and Sumner, 2005) and will be inconsistent with the claim that languages are activated differently in language comprehension and speech production tasks.

How stimulus valence affects switch costs when univalent and bivalent stimuli are randomly intermixed also has implications for theories of the switch cost asymmetry in speech production. The outcomes of Experiments 1 and 2 are consistent with accounts that attribute the switch cost asymmetry to competition between languages (Grainger and Beauvillain, 1987; Grainger and Dijkstra, 1992; Green, 1998; Meuter and Allport, 1999; Thomas and Allport, 2000; Orfanidou and Sumner, 2005; Peeters et al., 2014). One type of account attributes the switch cost asymmetry to competition between language schemas (e.g., Von Studnitz and Green, 1997; Green, 1998; Meuter and Allport, 1999; Thomas and Allport, 2000; Orfanidou and Sumner, 2005). According to these accounts, each language is associated with a language task schema. Successful speech production in one language requires the activation of the response language and the inhibition of the non-response language, which persists involuntarily when switching languages. Performance on a switch trial is slowed by having to overcome the inhibition required to respond in the appropriate language on the previous trial, yielding a switch cost. The cost of overcoming the prior inhibition of the currently relevant language is a function of the relative strength of the two languages. If the languages are unbalanced in strength, then naming an item in L2 requires strong(er) inhibition of L1, therefore switching to L1 will yield a large(er) cost because the time to overcome the strong inhibition of L1 from the previous language schema is longer. In contrast, naming an item in L1 only requires weak(er) inhibition of the L2 language, therefore the time to overcome L2 inhibition is shorter when switching to L2^6^.

A second type of account attributes the switch cost asymmetry to competition within the lexicon (Grainger and Beauvillain, 1987; Dijkstra and Van Heuven, 1998; Van Heuven et al., 1998; Peeters et al., 2014). According to these accounts, lexical representations are inhibited without the need for language task schemas and switch costs can be explained by mechanisms entirely within the language system. Here, greater inhibition of representations in L1 is required, in order to name an item in L2, yielding larger switch costs when switching back to L1 (Grainger and Dijkstra, 1992; Van Heuven et al., 1998; Grainger et al., 2010; Peeters et al., 2014).

Researchers investigating the locus of switch costs have repeatedly argued that if the cost of switching languages arises within the language system (as suggested by Grainger and colleagues), then switch costs should be reduced for stimuli with language specific orthography because they will differentially activate lexical representations in the two languages thereby reducing competition (Thomas and Allport, 2000; Orfanidou and Sumner, 2005; Peeters et al., 2014). The observation that switch costs are not reduced for univalent stimuli with language specific orthography in lexical decision when the univalent and bivalent stimuli are randomly intermixed has been used to support the claim that the control processes involved in language switching are outside the language system (Von Studnitz and Green, 1997; Thomas and Allport, 2000; Orfanidou and Sumner, 2005). Thus, the observation that stimulus valence affects switch costs, including the switch cost asymmetry when univalent and bivalent stimuli are intermixed (and therefore insensitive to context) in speech production would be inconsistent with the claim that the switch cost asymmetry arises from competition outside the language system, between language task schemas (Grainger and Beauvillain, 1987; Green, 1998; Meuter and Allport, 1999; Orfanidou and Sumner, 2005; Grainger et al., 2010; Peeters et al., 2014).

### Method

#### Participants

Forty-two undergraduate students at Trent University participated in return for credit in an eligible psychology course. These participants met the same criteria as participants in Experiments 1B and 2B. All participants had normal or corrected to normal vision.

#### Stimuli

The stimuli were the same numerals (bivalent) and number words (univalent) as Experiments 1B and 2B.

#### Apparatus

The apparatus was the same as Experiments 1B and 2B.

#### Procedure

The procedure was the same as Experiment 2 (alternating runs), except that the value of a target stimulus and its form (numeral vs. number word) were determined randomly on each trial (mixed blocks).

### Results

The present study did not include experiment as a factor. In all other ways the data were analyzed in the same way as Experiments 1 and 2. Mean response latencies and percentage error are displayed in Table 4.

**Table 4:** **Mean RT (ms) and percentage error as a function of stimulus type (numerals vs. words) and language (L1 vs. L2) and trial type (switch vs. non-switch) in Experiment 3**.

	**Numerals**		**Words**	
	**L1**	**L2**	**L1**	**L2**
**RESPONSE TIME**				
Switch	637	644	583	575
Non-switch	553	602	534	544
Cost	84	42	49	31
**PERCENTAGE ERROR**				
Switch	8.1	10.0	2.5	1.5
Non-switch	3.0	5.6	0.8	0.5
Cost	5.1	4.4	1.7	1.0

#### RT

Prior to analyzing the RT data, trials with incorrect responses (3.97%) or voice-key errors (2.27%) were removed. RTs for correct responses were subjected to the same recursive trimming procedure used in Experiments 1 and 2 (Van Selst and Jolicoeur, 1994). This resulted in the removal of an additional 2.35% of the data.

There was a main effect of stimulus type where subjects took 50 ms longer to respond to numerals compared to number words, *F*_(1, 41)_ = 79.3, *p* < 0.001, *MSE* = 2629, η_p_^2^ = 0.66. The effect of stimulus type was smaller than when stimulus valence was manipulated between blocks in Experiment 1 [*F*_(1, 78)_ = 126.76, *p* < 0.001, *MSE* = 4291, η_p_^2^ = 0.62] and Experiment 2 [*F*_(1, 74)_ = 50.23, *p* < 0.001, *MSE* = 7108, η_p_^2^ = 0.40]^5^.

Consistent with our participants being unbalanced bilinguals, there was a main effect of language where responses in L1 were 14 ms faster than responses in L2 [*F*_(1, 41)_ = 5.90, *p* < 0.05, *MSE* = 2911, η_p_^2^ = 0.13]. The L1 advantage was modulated by Stimulus Type, being larger for numerals (28 ms) than for number words (1 ms) [*F*_(1, 41)_ = 18.8, *p* < 0.001, *MSE* = 854, η_p_^2^ = 0.32], as was observed for blocked list presentation.

There was a main effect of trial type where switch trials were 51 ms slower than non-switch trials, *F*_(1, 41)_ = 229.6, *p* < 0.001, *MSE* = 965, η_p_^2^ = 0.85. Inconsistent with lexical decision (Von Studnitz and Green, 1997; Thomas and Allport, 2000; Orfanidou and Sumner, 2005), the switch costs were larger for (bivalent) numerals (63 ms) than for (univalent) number words (40 ms), *F*_(1, 41)_ = 68.7, *p* < 0.001, *MSE* = 159, η_p_^2^ = 0.63. The magnitude of the effect did not differ from those reported in Experiments 1 (*F* < 1) and 2 (*F* < 1) where stimulus valence was blocked^5^.

As expected, there was an interaction between language and trial type where the cost of switching languages was larger for L1 (66 ms) than for L2 (36 ms), replicating the switch cost asymmetry [e.g., Meuter and Allport, 1999; *F*_(1, 41)_ = 49.6, *p* < 0.001, *MSE* = 383, η_p_^2^ = 0.55]. Finally, the three way interaction between Trial Type, Language and Stimulus Type was significant, indicating that the asymmetry was smaller for the number words (18 ms) compared to the numerals (42 ms), *F*_(1, 41)_ = 8.04, *p* < 0.01, *MSE* = 356, η_p_^2^ = 0.16.

#### Percent error

There was nothing in the error data that compromised the interpretation of the RT data. There was a main effect of Stimulus Type where more errors were made for numerals (6.7%) compared to number words (1.3%), [*F*_(1, 41)_ = 97.1, *p* < 0.001, *MSE* = 24.8; η_p_^2^ = 0.70]. There was a main effect of language where more errors were made in L2 than L1 [*F*_(1, 41)_ = 4.68, *p* < 0.05, *MSE* = 10.7; η_p_^2^ = 0.10]. There was an interaction between Language and Stimulus Type where the effect of language was larger for numerals than for number words, *F*_(1, 41)_ = 22.3, *p* < 0.001, *MSE* = 7.97; η_p_^2^ = 0.35.

There was a main effect of Trial Type where more errors were made on switch (5.5%) than on non-switch (2.5%) trials, [*F*_(1, 41)_ = 87.2, *p* < 0.001, *MSE* = 8.80, η_p_^2^ = 0.68]. There was no interaction between Trial Type and Language, *F*_(1, 41)_ = 2.28, *MSE* = 4.58, η_p_^2^ = 0.05. However, there was an interaction between Trial Type and Stimulus Type where the switch cost was larger for numerals (4.7%) than for words (1.3%), *F*_(1, 41)_ = 48.2, *p* < 0.001, *MSE* = 4.96, η_p_^2^ = 0.54. No other effects approached significance.

### Discussion

Stimulus valence modulated overall switch costs and the switch cost asymmetry in the present experiment, despite the univalent and bivalent stimuli being presented in the same block of trials. The observation that stimulus valence influences switch costs irrespective of context during naming but not lexical decision is consistent with languages being activated differently and suggests that there are fundamental differences in how languages are controlled in comprehension and production tasks.

The present results are also inconsistent with accounts of language switching that place the control mechanisms entirely outside the language system such as the language task schema account of language switching, which predicts that stimulus valence should not affect switch costs under mixed list conditions (Thomas and Allport, 2000; Orfanidou and Sumner, 2005). The outcome of the present experiment is, however, consistent with within-language accounts of the switch cost asymmetry, which predict that the switch cost asymmetry should be affected by stimulus valence irrespective of context (Thomas and Allport, 2000; Orfanidou and Sumner, 2005; Finkbeiner et al., 2006; Peeters et al., 2014).

## General discussion

The standard account of the switch cost asymmetry in unbalanced bilinguals is that it arises from differences in the relative strength of the two languages. This account predicts that a switch cost asymmetry should arise in any language task when the strength of the two languages is unbalanced. Inconsistent with this account, the switch cost asymmetry is observed in speech production tasks but not in comprehension tasks, such as lexical decision and semantic categorization (see Table 1). Experiments 1 and 2 ruled out the possibility that these differences were due to two methodological factors (stimulus valence and switch predictability) that are confounded with the type of task. Experiment 3 demonstrated that languages are activated or operate differently for language comprehension and speech production tasks. Therefore, the present data converge on the claim that there are important task-related differences in how languages are controlled. Indeed, there are now three indicators that switch costs differ in lexical decision and speech production. First, switch costs are symmetric in the lexical decision task (e.g., Von Studnitz and Green, 1997; Thomas and Allport, 2000; Orfanidou and Sumner, 2005) and asymmetric in the speech production task (e.g., Meuter and Allport, 1999; Costa and Santesteban, 2004; Costa et al., 2006; Macizo et al., 2012), even when univalent stimuli (Experiments 1 and 2) and predictable switches are used (Experiment 2) so as to match the conditions usually observed in comprehension tasks. Second, switch costs are not affected by stimulus valence in mixed list contexts in lexical decision (Von Studnitz and Green, 1997; Thomas and Allport, 2000; Orfanidou and Sumner, 2005), but are in speech production (Experiment 3). Finally, stimulus valence has a larger impact for L1 than for L2 thereby reducing the switch cost asymmetry in speech production, but not in lexical decision (Thomas and Allport, 2000; Orfanidou and Sumner, 2005).

The present experiments are also consistent with at least some of the switch cost asymmetry arising from processing within the language system (Grainger and Beauvillain, 1987; Peeters et al., 2014). A switch cost asymmetry was observed for univalent number words in Experiments 1 and 2 when a single response selection criterion could be used, inconsistent with the switch cost asymmetry arising at the level of response selection as suggested by Finkbeiner et al. (2006; see also Peeters et al., 2014). Inconsistent with the switch cost asymmetry arising from competition between language schemas the switch cost asymmetry was affected by stimulus valence in Experiment 3 where the univalent and bivalent stimuli were randomly intermixed. According to language schema accounts switch costs should not be affected by factors that affect processing within the language system such as language specific orthography (Meuter and Allport, 1999; Thomas and Allport, 2000), especially under mixed list conditions (Orfanidou and Sumner, 2005). Both of these outcomes are predicted by within-language accounts of the switch cost asymmetry in which univalent stimuli either (1) directly activate a language node that specifies the appropriate language or (2) lead to stronger activation of lexical entries in the appropriate language by way of having a direct match in only one lexicon (this is not to say that do not activate lexical entries in the other language, e.g., Kroll et al., 2013).

### Why are asymmetric switch costs not observed in lexical decision?

The present experiments suggest that there are important differences in how languages are controlled during speech production and language comprehension tasks, and that at least some of these differences arise in the lexicon. Here, we propose that language switch costs arise from (at least) three sources: (1) language task schema activation / competition that affects response selection and initiation (Von Studnitz and Green, 1997; Thomas and Allport, 2000; Orfanidou and Sumner, 2005), (2) early activation of the language task schema (Orfanidou and Sumner, 2005) or language nodes (Grainger et al., 2010; Peeters et al., 2014) using stimulus attributes, and (3) within language activation/competition. Language task schemas specify which language is to be used and the specific configuration of the language system that is used to perform the language task (i.e., lexical decision, semantic categorization, speech production, etc.). According to this account, lexical decision, semantic categorization, and speech production tasks differ in terms of the specific lexical/semantic systems (e.g., orthography, phonology, semantics, syntax, etc.) required for task performance (see Green, 1998; Von Studnitz and Green, 2002). This information is specified as part of the language task schema. For instance, semantic categorization and lexical decision are often conceptualized as comprehension tasks because of a greater reliance on orthographic and semantic systems (e.g., Van Heuven et al., 1998; Peressotti et al., 2003; Yap et al., 2011) whereas naming is conceived as a production task because of its dependence on retrieving a representation for output (Meuter and Allport, 1999).

Here, we hypothesize that whether switch costs are symmetric or asymmetric in unbalanced bilinguals depends on both the relative strength of the language task schema and the specific levels of the language system specified as part of the language schema (e.g., semantics vs. phonology). Given the dependence of speech production tasks on phonological processing, we hypothesize that phonological processing is particularly susceptible to interference from activated entries in competing languages. Consistent with this possibility, generating a phonological code from print is known to require central attention, whereas orthographic processing does not (Reynolds and Besner, 2006).

Evidence that at least some of the switch cost asymmetry arises from competition within the language system does not preclude that some of the switch cost asymmetry arises outside the language system. Therefore, the observation that asymmetric switch costs persisted for univalent items could either be due to competition between phonological entries, despite the use of univalent number words, or it could be due to additional sources. Here, we consider two additional sources outside the lexicon that could give rise to the residual switch cost asymmetry, namely (1) response execution, and (2) language task schemas.

Unlike lexical decision, where the responses are likely equally novel for both L1 and L2, unbalanced bilinguals have more practice speaking in L1 than in L2. Therefore, it could be the case that there is competition between the processes involved in articulation or setting the parameters of a self-speech monitoring mechanism that checks one's own speech for errors or other problems. Although the present study does not rule out these possibilities, there is some evidence in the literature that suggests that the switch cost asymmetry does not arise from processes involved in response execution. For instance, if a switch cost asymmetry arises from competition between unbalanced response schemas, then a switch cost asymmetry should be observed in speech production whenever the languages are unbalanced in strength. Inconsistent with this prediction, the switch cost asymmetry is often absent for highly proficient bilinguals when switching between unbalanced languages (Costa and Santesteban, 2004; Costa et al., 2006; Martin et al., 2013). Furthermore, a switch cost asymmetry is not observed for unbalanced bilinguals in speech production when the languages switches are voluntary (Gollan and Ferreira, 2009). The switch cost asymmetry is not consistently observed for unbalanced bilinguals when the sequence of language switches is determined by patterns maintained in memory (Declerck et al., 2012). These latter approaches differ from cued studies in terms of how language selection takes place, but not how responses are executed. Taken together with the present findings, these studies suggest that very little, if any, of the switch cost asymmetry arises from response execution processes in unbalanced bilinguals.

A second source outside the lexicon that could give rise to the residual switch cost asymmetry is competition between language task schemas. If the relative strength of the language task schema depends on experience with the configuration of the language system required for task performance and stimulus response mapping (at least in the case of lexical decision), then for unbalanced bilinguals this should result in more experience with the configuration of the language system required for speech production in L1 than L2. Thus, it is possible that the language task schemas will be unbalanced in speech production for unbalanced bilinguals. However, this should be less pronounced in a task like lexical decision, which, as noted by Thomas and Allport (2000), requires “the introduction of arbitrary, task-specific components to the use of the bilingual's languages” (p. 62). As such, the language task schemas will be balanced in lexical decision, and other tasks where the system configuration is novel, yielding symmetric switch costs.

Although we postulate that at least part of the switch cost asymmetry arises from competition between phonological representations, which may be more sensitive to competition from entries in other languages, another possibility is that sensitivity to competition is tied to a more general difference between the organization of input and output lexicons. Models of language processing often specify separate input and output orthographic and phonological lexicons (e.g., Coltheart et al., 2001; Coltheart, 2004). To the best of our knowledge, task type (comprehension vs. production) has been confounded with the type of internal representation required for task completion (e.g., orthographic vs. phonological). It is therefore unclear whether differences in performance are a consequence of how input and output systems are controlled, or whether there are differences in how orthographic and phonological systems are interconnected in bilingual speakers. For instance, Grainger et al.'s (2010) developmental version of the Bilingual Interactive Activation (BIA) model attributes switch costs to different mechanisms in comprehension (which have used words as stimuli) and production tasks (which have used bivalent stimuli such as numerals and pictures). In their view, univalent words exogenously activate the appropriate language node in comprehension tasks, which selectively enhances processing in one language relative to the other language. In speech production, the use of bivalent stimuli requires top-down control over the language node. According to this account the switch cost asymmetry arises in speech production because endogenous activation of the language node yields greater inhibition of the lexical representations for L1 than for L2. The observation that univalent stimuli reduce switch costs and the switch cost asymmetry can be explained by univalent stimuli exogenously activating the language nodes, thereby reducing the contribution of endogenous control processes that give rise to the asymmetry. One issue that this account has difficulty explaining is the persistence of a switch cost asymmetry in Experiments 2 and 3 where predictable switches between languages occurred. In these experiments, the average response-stimulus interval (RSI) was long (756 ms in Experiment 2A, 497 ms in Experiment 2B, and 706 ms in Experiment 3)^7^. A switch cost asymmetry has also been reported for unbalanced bilinguals using predictable switches and an RSI of 1500 ms by Jackson et al. (2001). This is problematic for the endogenous control account of the switch cost asymmetry because evidence suggests that at RSIs beyond 500 ms, switch costs are primarily driven exogenously by the stimulus itself when switches are predictable (Rogers and Monsell, 1995). Therefore, the persistence of the switch cost asymmetry at long RSIs suggests that the switch cost asymmetry is not due to endogenous control. Converging evidence comes from studies examining the role of advance preparation (endogenous control) under conditions where the language switches are random (as in Experiment 1). In these studies, the role of endogenous processes in the switch cost asymmetry assessed by examining how it is affected by the cue-stimulus interval (CSI). These studies have reported inconsistent effects of CSI on the switch cost asymmetry (e.g., Philipp et al., 2007; Verhoef et al., 2009; Declerck et al., 2012; Fink and Goldrick, 2015). Consequently, there seems to be little evidence to support the claim that the switch cost asymmetry in speech production is driven purely by top-down endogenous control processes.

### Why are language comprehension and speech production affected differently by stimulus valence?

Accounts of language switching also need to explain why stimulus valence does not affect switch costs in the lexical decision task when the univalent and bivalent stimuli are randomly intermixed in a single block of trials. Here, we hypothesize that there was no reduction in the switch costs for univalent items in lexical decision because switch costs in this task have been largely due to processes outside the lexicon, such as competition between language task schemas (Von Studnitz and Green, 1997; Thomas and Allport, 2000). To date, the words used in lexical decision have been unique to one language (and therefore arguably univalent), yet stimulus valence was further defined according to the presence or absence of language specific orthographic cues (e.g., combinations of letters). If switch costs arising from within the language system are largely limited to competition between orthographic-lexical representations in lexical decision (as opposed to phonological-lexical representations in speech production), then this raises the possibility that switch costs arising within the language system were already minimized by the language specific nature of the words, and therefore were not reduced further by using words with language specific orthography. Support for this hypothesis comes from evidence that the majority of the switch cost in lexical decision is due to changing the response selection criteria (Von Studnitz and Green, 1997; Thomas and Allport, 2000).

### Non-task associated differences

Finally, it is always possible that there are other task-associated differences that become candidates for differences in language switching across tasks. For instance, lexical decision includes the use of non-words, which were not included in the present experiments. There is evidence from research on visual word recognition and reading aloud that the presence of non-words in a context can change how sublexical and lexical information affect one another. For instance, stimulus quality and word frequency yield additive effects in lexical decision (Yap and Balota, 2007). In contrast, stimulus quality and word frequency interact in reading aloud (O'Malley et al., 2007) unless non-words are added to the list context, in which case their effects are additive (O'Malley and Besner, 2008). This suggests that the presence of non-words could change how lexical information is activated (see also Thomas and Allport, 2000). In this instance, the presence of non-words is unlikely to be the driving factor that determines whether switch costs are symmetric, because switch costs are symmetric in semantic categorization, where non-words are not part of the stimulus set. This is not to say, however, that there are no other differences. At present, however, we believe that there is sufficient evidence to justify further investigation into task related differences in bilingual language switching.

### Implications for highly proficient bilinguals

The switch cost asymmetry is not usually observed when highly proficient bilinguals (e.g., those that are balanced in L1 and L2), switch between established languages (L1, L2, or L3), but is observed when they switch between languages of low proficiency (L3, L4, or a new language; Costa and Santesteban, 2004; Costa et al., 2006; Martin et al., 2013). In order to explain this pattern, Costa and colleagues suggested that highly proficient bilinguals have available two mechanisms for selecting a language (1) a language-specific selection mechanism and (2) within-language inhibitory control. The language-specific selection mechanism operates when switching between languages with established lexicons by setting different criteria for lexical selection in each language. This mechanism does not change how the languages operate; instead it operates on the output of the language system. Switching between language-specific selection criteria is independent of language strength and therefore yields symmetric switch costs. Inhibitory control operates when one of the lexicons is not well formed so that a language specific selection criterion cannot be established. In this instance inhibitory mechanisms affect lexical representations in the dominant language (e.g., L1) proportional to language strength yielding asymmetric switch costs. The present findings are consistent with unbalanced bilinguals using an inhibitory mechanism that affects processing within a language system. Interestingly, this dual process account of language switching in highly proficient bilinguals could be tested by examining how switch costs are affected by stimulus valence in mixed list conditions as in Experiment 3. If stimulus valence affects switch costs arising from within the language system, then stimulus valence should interact with switch costs when highly proficient bilinguals switch between low proficiency languages (e.g., L3 and L4) because the within-language inhibitory mechanism will be operating. In contrast, stimulus valence should not affect switch costs when highly proficient bilinguals switch between established languages (e.g., L1 and L2) where only the language-specific selection mechanism is operating.

## Conclusion

Experiments 1 and 2 demonstrated that in speech production the asymmetric switch costs are not dependent on the presence of bivalent stimuli, nor on switch predictability. Experiment 3 demonstrated that the effects of stimulus valence affects switch costs and the asymmetric switch cost during speech production, despite numerous demonstrations that this is not the case in lexical decision (Thomas and Allport, 2000; Orfanidou and Sumner, 2005). Furthermore, the modulation of the switch cost asymmetry by stimulus valence and the persistence of the switch cost for univalent items is best accounted for by theories of language switching that posit a role for competition within the language system. In particular, we suggest that the switch cost asymmetry arises because a component of the language system required for speech production (namely phonology) is particularly susceptible to interference from the competing language. The observation that speech production continues to reveal a different pattern of switch costs compared to comprehension tasks suggests that future research needs to continue to examine the similarities and differences in performance across tasks.

### Conflict of interest statement

The authors declare that the research was conducted in the absence of any commercial or financial relationships that could be construed as a potential conflict of interest.

## Footnotes

^1^Here we chose to examine how the switch cost asymmetry in speech production was affected by factors used in lexical decision (univalent stimuli, predictable switches) for two reasons. First, incorporating the stimuli used in lexical decision experiments (written words) into a speech production task was feasible, whereas incorporating the bivalent stimuli used in naming (numerals and objects) into lexical decision and/or semantic categorization looks quite complicated. Indeed we could not think of an appropriate forced choice task with stimuli such as pictures and numerals, which require language switches. For example making parity judgments to numerals can be done without switching languages. The same is true if bivalent words (i.e., homographs) are used given that on a switch trial the homograph would yield a correct answer whether the subject switched languages or not (see Von Studnitz and Green, 1997; Thomas and Allport, 2000). Clearly the impossibility of pairing of bivalent stimuli with forced choice tasks also rendered a direct comparison between speech production and decision tasks problematic. Second, we chose the naming task since evidence suggests that it is more appropriate for investigating the processes involved in lexical processing. This is mainly due to the fact that the naming task avoids complications from decision processes observed in alternative forced choice tasks (e.g., Balota and Chumbley, 1984).

^2^Two types of bilinguals were used in the present studies: English-French bilinguals and Italian-English bilinguals. The purpose of using two different groups of bilinguals was to assess whether the outcome of the experiments generalized across language combinations and whether having English (a language with highly inconsistent spelling-to-sound correspondences) as L1 or L2 affected performance during language switching. As will be seen below, there were no systematic differences between the two types of bilinguals.

^3^There may be additional relevant differences other than predictability. For instance, Altmann (2007) has noted that switch costs are generally larger in the alternating runs paradigm because the switch costs in this paradigm include costs associated with switching and costs associated with decoding the cue. Further, the use of long response-stimulus intervals (RSI) may eliminate endogenous contributions to the switch cost (c.f. Rogers and Monsell, 1995). Assessing whether the switch cost asymmetry is eliminated in the alternating runs paradigm as a whole therefore tests whether the absence of an asymmetry is due to any number of known and unknown differences between these two methods.

^4^The use of an AABB trial pattern increases the probability of a switch from 0.3 in Experiment 1 to 0.5 in Experiment 2. The observation of a switch cost asymmetry for the univalent stimuli in the present experiment suggests that the probability of a switch is not a determining factor for when a switch cost asymmetry is observed.

^5^All comparisons across studies (e.g., Experiment 1 vs. Experiment 2) were conducted using a mixed model ANOVA with Language (L1 vs. L2), Stimulus Type (numerals vs. words) and Trial Type (switch vs. non-switch) as repeated factors and Study (e.g., Experiment 1 vs. Experiment 2) as a between subjects factor.

^6^Although inhibition is often invoked to explain asymmetric switch costs (especially in language switching), it may not be required. For instance task set activation can also account for the data (see Koch et al., 2010, for a more elaborate discussion).

^7^The RSI consisted of how long it took the researcher to code a subject's vocal response and a 250 delay at the beginning of the trial next trial.

## References

[B1] Abutalebi J. Green D. (2007). Bilingual language production: the neurocognition of language representation and control. J. Neurolinguist. 20, 242–275. 10.1016/j.jneuroling.2006.10.003

[B2] Allport A. Styles E. A. Hsieh S. (1994). Shifting intentional set: exploring the dynamic control of tasks, in Conscious and Nonconscious Information Processing: Attention and Performance XV, eds Umiltà C. Moscovitch M. (Cambridge, MA: MIT Press), 421–452.

[B3] Altmann E. M. (2007). Comparing switch costs: alternating runs and explicit cuing. J. Exp. Psychol. Learn. Mem. Cogn. 33, 475–483. 10.1037/0278-7393.33.3.475 17470001

[B4] Balota D. A. Chumbley J. I. (1984). Are lexical decisions a good measure of lexical access? The role of word frequency in the neglected decision stage. J. Exp. Psychol. Hum. Percept. Perform. 10, 340–357. 10.1037/0096-1523.10.3.340 6242411

[B5] Christoffels I. K. Firk C. Schiller N. O. (2007). Bilingual language control: an event-related potential study. Brain Res. 1147, 192–208. 10.1016/j.brainres.2007.01.137 17391649

[B6] Chumbley J. I. Balota D. A. (1984). A word's meaning affects the decision in lexical decision. Mem. Cognit. 12, 590–606. 10.3758/BF03213348 6533428

[B7] Coltheart M. (2004). Are there lexicons? Q. J. Exp. Psychol. 57, 115–1171. 10.1080/02724980443000007 15513241

[B8] Coltheart M. Rastle K. Perry C. Langdon R. Ziegler J. (2001). DRC: a dual route cascaded model of visual word recognition and reading aloud. Psychol. Rev. 108, 204–256. 10.1037/0033-295X.108.1.204 11212628

[B9] Costa A. Santesteban M. (2004). Lexical access in bilingual speech production: evidence from language switching in highly proficient bilinguals and L2 learners. J. Mem. Lang. 50, 491–511. 10.1016/j.jml.2004.02.002

[B10] Costa A. Santesteban M. Ivanova I. (2006). How do highly proficient bilinguals control their lexicalization process? Inhibitory and language-specific selection mechanisms are both functional. J. Exp. Psychol. 32, 1057–1074. 10.1037/0278-7393.32.5.1057 16938046

[B11] Damian M. F. (2004). Asymmetries in the processing of Arabic digits and number words. Mem. Cognit. 32, 164–171. 10.3758/BF03195829 15078053

[B12] Declerck M. Koch I. Philipp A. M. (2012). Digits vs. pictures: the influence of stimulus type on language switching. Bilingualism 15, 896–904. 10.1017/S1366728912000193

[B13] Dijkstra A. Van Heuven W. J. B. (1998). The BIA- model and bilingual word recognition, in Localist Connectionist Approaches to Human Cognition, eds Grainger J. Jacobs A. (Hillsdale NJ: Erlbaum), 189–225.

[B14] Fink A. Goldrick M. (2015). Pervasive benefits of preparation in language switching. Psychon. Bull. Rev. 22, 808–814. 10.3758/s13423-014-0739-6 25257712

[B15] Finkbeiner M. Almeida J. Janssen N. Caramazza A. (2006). Lexical selection in bilingual speech production does not involve language suppression. J. Exp. Psychol. Learn. Mem. Cogn. 32, 1075–1089. 10.1037/0278-7393.32.5.1075 16938047

[B16] Gollan T. H. Ferreira V. S. (2009). Should I stay or should I switch? A cost-benefit analysis of voluntary language switching in young and aging bilinguals. J. Exp. Psychol. Learn. Mem. Cogn. 35, 640–665. 10.1037/a0014981 19379041 PMC2864120

[B17] Grainger J. Beauvillain C. (1987). Language blocking and lexical access in bilinguals. Q. J. Exp. Psychol. 39A, 295–319. 10.1080/14640748708401788

[B18] Grainger J. Dijkstra T. (1992). On the representation and use of language information in bilinguals, in Cognitive Processing in Bilinguals, ed Harris R. J. (Amsterdam: Elsevier), 207–220.

[B19] Grainger J. Midgley K. Holcomb P. J. (2010). Re-thinking the bilingual interactive-activation model from a developmental perspective (BIA-d), in Language Acquisition Across Linguistic and Cognitive Systems, eds Kail M. Hickmann M. (New York, NY: Benjamins), 267–284.

[B20] Green D. W. (1998). Mental control of the bilingual lexico-semantic system. Bilingualism 1, 67–81. 10.1017/S1366728998000133

[B21] Humphreys G. W. Price C. J. Riddoch M. J. (1999). From objects to names: A cognitive neuroscience approach. Psychol. Res. 62, 118–130. 10.1007/s004260050046 10472198

[B22] Jackson G. M. Swainson R. Cunnington R. Jackson S. R. (2001). ERP correlates of executive control during repeated language switching. Bilingualism 4, 169–178. 10.1017/s1366728901000268

[B23] Jackson G. M. Swainson R. Mullin A. Cunnington R. Jackson S. R. (2004). ERP correlates of a receptive language-switching task. Q. J. Exp. Psychol. 57, 223–240. 10.1080/02724980343000198 14742175

[B24] Kiesel A. Steinhauser M. Wendt M. Falkenstein M. Jost K. Philipp A. M.. (2010). Control and interference in task switching - A review. Psychol. Bull. 136, 849–874. 10.1037/a0019842 20804238

[B25] Koch I. Gade M. Schuch S. Philipp A. M. (2010). The role of inhibition in task switching – A review. Psychon. Bull. Rev. 17, 1–14. 10.3758/PBR.17.1.1 20081154

[B26] Kroll J. F. Gullifer J. W. Rossi E. (2013). The multilingual lexicon: the cognitive and neural basis of lexical comprehension and production in two or more languages. Annu. Rev. Appl. Linguist. 33, 102–127. 10.1017/S0267190513000111

[B27] Macizo P. Bajo T. Paolieri D. (2012). Language switching and language competition. Second Lang. Res. 28, 131–149. 10.1177/0267658311434893

[B28] Martin C. D. Strijkers K. Santesteban M. Escera C. Hartsuiker R. J. Costa A. (2013). The impact of early bilingualism on controlling a language learned late: an ERP study. Front. Psychol. 4:815. 10.3389/fpsyg.2013.00815 24204355 PMC3817381

[B29] Meuter R. F. I. Allport A. (1999). Bilingual language switching in naming: asymmetrical costs of language selection. J. Mem. Lang. 40, 25–40. 10.1006/jmla.1998.2602

[B30] Ministry of Education Training (1998). The Ontario Curriculum: Core French, Grades 4-8. Available online at: http://www.edu.gov.on.ca/eng/curriculum/elementary/fsl.html

[B31] Ministry of Education Training (1999). The Ontario Curriculum Grades 9 and 10: Core, Extended, and Immersion French. Available online at: http://www.edu.gov.on.ca/eng/curriculum/secondary/fsl.html

[B32] O'Malley S. Besner D. (2008). Reading aloud: qualitative differences in the relation between stimulus quality and word frequency as a function of context. J. Exp. Psychol. Learn. Mem. Cogn. 34, 1400–1411. 10.1037/a0013084 18980404

[B33] O'Malley S. Reynolds M. G. Besner D. (2007). Qualitative differences between the joint effects of stimulus quality and word frequency in reading aloud and lexical decision: extensions to Yap and Balota. J. Exp. Psychol. Learn. Mem. Cogn. 33, 451–458. 10.1037/0278-7393.33.2.451 17352625

[B34] Orfanidou E. Sumner P. (2005). Language switching and the effects of orthographic specificity and response repetition. Mem. Cogn. 33, 355–369. 10.3758/BF03195323 16028589

[B35] Peeters D. Runnqvist E. Bertrand D. Grainger J. (2014). Asymmetrical switch costs in bilingual language production induced by reading words. J. Exp. Psychol. Learn. Mem. Cogn. 40, 284–292. 10.1037/a0034060 23957363

[B36] Peressotti F. Cubelli R. Job R. (2003). On recognizing proper names: the orthographic cue hypothesis. Cogn. Psychol. 47, 87–116. 10.1016/S0010-0285(03)00004-5 12852936

[B37] Perry C. Ziegler J. C. Zorzi M. (2007). Nested incremental modeling in the development of computational theories: the CDP+ model of reading aloud. Psychol. Rev. 114, 273–315. 10.1037/0033-295X.114.2.273 17500628

[B38] Philipp A. M. Gade M. Koch I. (2007). Inhibitory processes in language switching: evidence from switching language-defined response sets. Eur. J. Cogn. Psychol. 19, 395–416. 10.1080/09541440600758812

[B39] Reynolds M. Besner D. (2006). Reading aloud is not automatic: processing capacity is required to generate a phonological code from print. J. Exp. Psychol. Hum. Percept. Perform. 32, 1303–1323. 10.1037/0096-1523.32.6.1303 17154774

[B40] Rogers R. D. Monsell S. (1995). Costs of a predictable switch between simple cognitive tasks. J. Exp. Psychol. 124, 207–231. 10.1037/0096-3445.124.2.207

[B41] Thomas M. S. C. Allport A. (2000). Language switching costs in bilingual visual word recognition. J. Mem. Lang. 43, 44–66. 10.1006/jmla.1999.2700

[B42] Treasury Board of Canada Secretariat (2012). Qualification Standards in Relation to Official Languages. Available one at: http://www.tbs-sct.gc.ca/gui/squn03-eng.asp

[B43] Vandierendonck A. Liefooghe B. Verbruggen F. (2010). Task switching: interplay of reconfiguration and interference control. Psychol. Bull. 136, 601–626. 10.1037/a0019791 20565170

[B44] Van Heuven W. J. B. Dijkstra A. Grainger J. (1998). Orthographic neighborhood effects in bilingual word recognition. J. Mem. Lang. 39, 458–483. 10.1006/jmla.1998.2584

[B45] Van Selst M. Jolicoeur P. (1994). A solution to the effect of sample size on outlier elimination. Q. J. Exp. Psychol. 47A, 631–650.

[B46] Verhoef K. Roelofs A. Chwilla D. J. (2009). Role of inhibition in language switching: evidence from event-related brain potentials in overt picture naming. Cognition 110, 84–99. 10.1016/j.cognition.2008.10.013 19084830

[B47] Von Studnitz R. E. Green D. W. (1997). Lexical decision and language switching. Int. J. Bilingualism, 1, 3–24. 10.1177/136700699700100102

[B48] Von Studnitz R. E. Green D. W. (2002). The cost of switching language in a semantic categorization task. Bilingualism 5, 241–251. 10.1017/S1366728902003036

[B49] Yap M. J. Balota D. A. (2007). Additive and interactive effects on response time distributions in visual word recognition. J. Exp. Psychol. Learn. Mem. Cogn. 33, 274–296. 10.1037/0278-7393.33.2.274 17352611

[B50] Yap M. J. Tan S. E. Pexman P. M. Hargreaves I. S. (2011). Is more always better? Effects of semantic richness, on lexical deision, speeded naming and semantic classification. Psychon. Bull. Rev. 18, 742–750. 10.3758/s13423-011-0092-y 21494916

